# Modeling and Performance Analysis of a Hybrid Forward Osmosis–Membrane Distillation System for Seawater Desalination

**DOI:** 10.3390/membranes16040142

**Published:** 2026-04-02

**Authors:** Zakaria Triki, Zineb Fergani, Hichem Tahraoui, Nassim Moula, Jie Zhang, Abdeltif Amrane, Farid Fadhilah, Amine Aymen Assadi

**Affiliations:** 1 Laboratory of Renewable Energies and Materials (LERM), University of Medea, Medea 26000, Algeria; 2 Laboratory of Biomaterials and Transport Phenomena (LBMPT), University of Medea, Medea 26000, Algeria; tahraoui.hichem@univ-medea.dz; 3 Fundamental and Applied Research in Animal and Health (FARAH), Department of Veterinary Management of Animal Resources, Faculty of Veterinary Medicine, University of Liege, 4000 Liege, Belgium; 4 School of Engineering, Merz Court, Newcastle University, Newcastle upon Tyne NE1 7RU, UK; 5 Ecole Nationale Supérieure de Chimie de Rennes, CNRS, ISCR—UMR6226, University Rennes, F-35000 Rennes, France; abdeltif.amrane@univ-rennes.fr; 6 College of Engineering, Imam Mohammad Ibn Saud Islamic University (IMSIU), Riyadh 11432, Saudi Arabia

**Keywords:** seawater desalination, forward osmosis, membrane distillation, modeling, performance analysis

## Abstract

Hybrid desalination systems that combine osmotic and thermal driving forces offer a promising route to improve water recovery and energy efficiency for high-salinity feedwaters where conventional processes face limitations. This study presents a comprehensive mathematical modeling framework and performance analysis of a hybrid forward osmosis–membrane distillation (FO-MD) system for seawater desalination. The novel contributions include: (1) a coupled heat, mass, and solute transport model that explicitly accounts for concentration polarization, temperature polarization, reverse salt flux, and their dynamic interactions through the draw solution loop; (2) a quantitative assessment of the synergistic regeneration effect, showing how MD maintains draw solution concentration and stabilizes FO performance over time; (3) systematic evaluation of parameter sensitivity to polarization effects; and (4) comparative energy analysis quantifying specific energy consumption relative to standalone processes. Model predictions were validated against published experimental data, showing good agreement for both FO and MD fluxes (R^2^ > 0.94). The MD flux increased from approximately 2–3 LMH at 30 °C to 17 LMH at 50 °C, confirming vapor pressure enhancement. FO water flux increased significantly with draw solution concentration from 0.2 to 1.1 M due to higher osmotic pressure differences. Time-dependent simulations of the integrated FO-MD system showed that MD regeneration reduces draw solution dilution by 60% compared to standalone FO, maintaining FO flux approximately 43% higher after 6 h of operation. Sensitivity analysis revealed that FO predictions are moderately sensitive to mass transfer coefficients (6–9% flux change for 20% parameter variation), while MD shows lower sensitivity to heat transfer coefficients (3–5%). Energy analysis indicates that FO-MD hybridization reduces thermal energy consumption by 15–40% compared to standalone MD, with specific energy consumption of 382 kWh/m^3^ (40.2 kWh/m^3^ primary energy equivalent) when using low-grade heat. The obtained results demonstrate that FO-MD hybridization enhances water recovery and operational stability compared to standalone processes, supporting its potential for energy-efficient desalination of high-salinity brines and industrial wastewaters where low-grade heat is available.

## 1. Introduction

The global shortage of freshwater resources, driven by population growth, industrial expansion, and climate change, has emerged as a major sustainability challenge in the twenty-first century. Desalination has consequently become an indispensable technology for augmenting water supplies in arid and coastal regions where conventional freshwater sources are insufficient [1,2].

Despite substantial technological progress, conventional desalination methods are still constrained by high specific energy consumption and environmental concerns related to brine management and dependence on fossil-derived energy. As the interconnection between water and energy security intensifies, growing attention has been directed toward the development of advanced desalination systems that combine high water recovery efficiency, reduced energy demand, and effective integration with renewable heat sources [3,4].

Among these technologies, reverse osmosis (RO) currently dominates the desalination market owing to its high efficiency and maturity. Nevertheless, RO faces several limitations, including high operating pressure, membrane fouling, limited salt rejection for highly concentrated feeds, and difficulties in achieving high brine concentration levels suitable for resource recovery [5,6]. Thermal processes such as multi-effect distillation (MED) and multi-stage flash (MSF) distillation, while capable of handling high salinity, require significant heat input and are constrained by scaling, corrosion, and maintenance costs [7,8,9]. Consequently, innovative low-energy hybrid desalination configurations that combine osmotic and thermal driving forces have gained increasing attention as sustainable alternatives.

Forward osmosis (FO) has emerged as an energy-efficient membrane process driven by osmotic pressure differences across a semi-permeable membrane [10,11]. FO offers several advantages, including low hydraulic pressure operation, reduced fouling propensity, and the ability to handle high-salinity feedwaters [12]. Nevertheless, its widespread application remains hindered by challenges associated with the regeneration of the draw solution and the relatively low permeate flux compared to pressure-driven systems [13]. On the other hand, membrane distillation (MD) is a thermally driven separation process that utilizes a hydrophobic microporous membrane and a temperature-induced vapor pressure gradient to facilitate mass transfer [14,15]. MD can efficiently utilize low-grade or renewable heat sources, making it particularly attractive for integration with solar or waste heat systems.

The integration of FO and MD in a hybrid configuration offers a synergistic approach that combines the osmotic separation efficiency of FO with the thermally driven regeneration capability of MD. In this hybrid system, FO concentrates on the feed solution while MD simultaneously regenerates the draw solution, effectively overcoming one of the key bottlenecks of standalone FO systems [16]. This coupling enables continuous operation, enhances overall water recovery, and significantly reduces specific energy consumption by utilizing low-grade or waste heat for MD regeneration. Furthermore, it enables the generation of highly concentrated brine streams that are suitable for mineral extraction, thus contributing to circular desalination and resource recovery frameworks [17].

Recently, the hybridization of FO and MD has gained substantial attention for the treatment of high-salinity and complex waste streams. Several studies have demonstrated that the integrated FO-MD system can achieve superior contaminant rejection and higher operational stability compared to either process alone. For instance, Zhou et al. [18] conducted a comprehensive optimization of the hybrid configuration by adjusting key operational parameters, including the feed and draw solution flow rates, draw solute concentration, and the inlet temperature of the MD unit. Their findings indicated that the optimized FO-MD system exhibited remarkable potential for efficiently treating hypersaline and hazardous wastewaters while maintaining stable flux performance.

Lu et al. [19] further integrated this configuration with ultrafiltration (UF) for oily water treatment. The three-channel architecture provides inherent flux balancing between the FO and MD sections and offers a compact system layout. Nevertheless, the increased thermal conduction across the shared channels may lead to additional heat loss, thereby reducing the overall energy efficiency. In addition, Nawaz et al. [17] experimentally evaluated FO-MD using real produced water streams, reporting stable fluxes, manageable fouling behavior, and high contaminant removal, while Mat Nawi et al. [20] highlighted the influence of draw solution temperature, concentration, and FO-MD equilibrium on flux performance.

Nevertheless, despite these promising results, research on FO-MD coupling remains relatively limited, particularly concerning the long-term stability of the draw solution and the mitigation of interfacial fouling and solute accumulation phenomena. Only a few studies have systematically addressed these aspects under realistic operating conditions, highlighting the need for further experimental and modeling investigations to better understand and optimize the coupled mass and heat transfer processes governing FO-MD hybrid systems.

To address these gaps, the present study develops a comprehensive mathematical model and performance evaluation framework for a hybrid FO-MD desalination process. The model accounts for the simultaneous transport of water, solutes, and heat within both modules and enables predictive simulations of system behavior under varying feed salinity, temperature gradients, and flow conditions. Parametric analyses are conducted to identify optimal operating regimes that maximize water flux and recovery while minimizing specific energy consumption. The findings of this work contribute to advancing scalable, energy-efficient, and resource-oriented desalination technologies that support sustainable water management.

## 2. System Description

**Figure 1:**
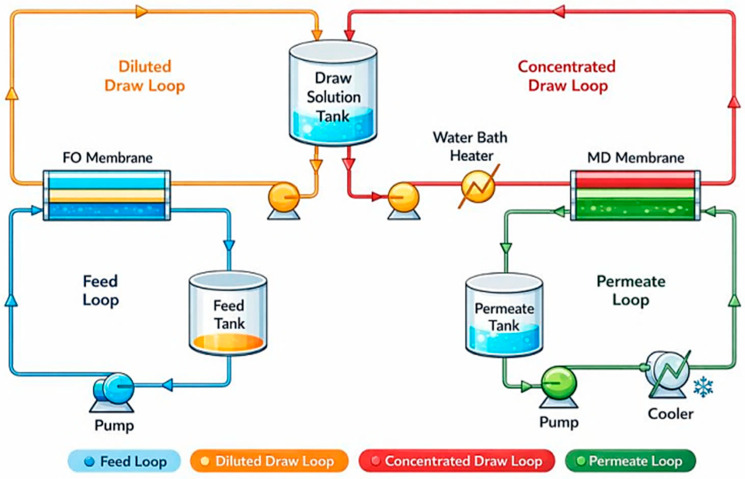
Schematic diagram of the FO-MD hybrid system.

The hybrid FO-MD system investigated in this study is designed to desalinate seawater by coupling osmotic-driven water extraction with thermal-driven draw solution regeneration. The system (Figure 1) consists of three hydraulically independent loops: a feed loop, a draw solution loop, and a permeate loop. These loops are interconnected through an FO membrane module and an MD membrane module, with a central draw solution tank serving as the interface between the two membrane processes.

In the FO subsystem, synthetic seawater is stored in a feed tank and circulated through the FO membrane module using a low-pressure pump. The feed solution flows on one side of the semi-permeable FO membrane, while a concentrated draw solution flows on the opposite side. Water permeation from the feed to the draw solution occurs due to the osmotic pressure difference, resulting in progressive dilution of the draw solution and concentration of the feed. The FO process operates under near-atmospheric pressure, which minimizes energy consumption and reduces membrane fouling compared to pressure-driven desalination processes such as reverse osmosis.

The diluted draw solution exiting the FO membrane is collected in a draw solution tank, which functions as a buffer to stabilize flow, concentration, and temperature variations between the FO and MD subsystems. From this tank, the diluted draw solution is pumped into the MD loop for regeneration. Before entering the MD membrane module, the draw solution is heated using a water bath heater to establish a sufficient vapor pressure gradient across the hydrophobic MD membrane.

Within the MD module, water vapor selectively evaporates from the heated draw solution, diffuses through the membrane pores, and condenses on the cooler permeate side. Non-volatile draw solutes are effectively rejected, enabling reconcentration of the draw solution. The reconcentrated draw solution is then returned to the draw solution tank and recirculated back to the FO membrane, completing the closed-loop operation of the draw solution.

The permeate produced by the MD process is collected in a permeate tank and circulated through a cooling unit to maintain a low permeate temperature and enhance vapor condensation. The recovered permeate represents the final product water of the system and is characterized by high purity due to the combined solute rejection mechanisms of FO and MD. Independent control of flow rates and temperatures in each loop allows systematic evaluation of system performance, including water flux, salt rejection, energy efficiency, and regeneration efficiency.

## 3. FO-MD Mathematical Model

In this study, the FO-MD hybrid system was modeled using steady-state membrane transport equations coupled through time-dependent draw solution mass and energy balances, resulting in a quasi-dynamic formulation for predicting flux evolution during operation.

### 3.1. Model Assumptions

The mathematical model has been developed based on the following assumptions:

- Mass and heat transfer are assumed one-dimensional and normal to the membrane surface; axial variations along the membrane length are neglected.
- Membrane properties (water and salt permeability, porosity, tortuosity, thickness, and thermal conductivity) are uniform and constant during operation.
- Water transport in the FO membrane is driven only by osmotic pressure difference, with negligible hydraulic pressure difference, and follows the solution-diffusion mechanism with constant permeability coefficients.
- External and internal concentration polarization (CP) effects are incorporated using analytical mass transfer expressions with constant mass transfer coefficients; reverse salt flux is included via a constant salt permeability coefficient, while ion-ion interactions are neglected.
- MD membrane pores are assumed completely non-wetted, and mass transfer occurs exclusively in the vapor phase, driven by vapor pressure differences induced by temperature gradients, following combined Knudsen-molecular diffusion.
- All draw solution solutes are assumed non-volatile and fully rejected by the MD membrane.
- The diluted draw solution exiting the FO module is perfectly coupled to the MD module, enabling matched overall water transfer rates between FO and MD.

### 3.2. FO Transport Model

FO transport is governed by osmotic-pressure-driven water permeation through a semi-permeable membrane, accounting for CP, reverse salt flux (RSF), and membrane fouling. The osmotic pressure of feed and draw solutions is given by the van’t Hoff equation [21]:

(1) π=ϕnCRT

where ϕ is the osmotic coefficient, n is the van’t Hoff coefficient, C is the molar concentration of the solute, R is the ideal gas constant, and *T* is the absolute temperature of the solution.

For NaCl solutions near 1 M, ϕ ≈ 0.93, resulting in an osmotic pressure reduction of approximately 5–10% compared to the ideal assumption.

The general FO water flux, including internal CP, external CP, and RSF coupling, is [22]:

(2) $$J_{w}=A\frac{\pi_{D,b}\exp\left(-J_{w}K\right)-\pi_{F,b}exp\left(\frac{J_{w}}{k}\right)}{1+\frac{B}{J_{w}}\left[exp\left(\frac{J_{w}}{k}\right)-\exp\left(-J_{w}K\right)\right]}$$

where:

A and B are the water and solute permeability coefficients, respectively.

$\pi_{D,b}$ and $\pi_{F,b}$ are the bulk osmotic pressures in draw and feed side solutions, respectively.

k and K are mass transfer and solute resistivity, respectively.

The mass transfer resistivity, k, is determined using the following equation:

(3) $$k=\frac{shD_{s}}{d_{h}}$$

where $D_{s}$ is the solute diffusion coefficient, $d_{h}$ is the hydraulic diameter of the channel, and sh  is the Sherwood number.

The solute resistivity for diffusion within the porous support layer, K, is defined by [23]:

(4) $$K=\frac{S}{D_{s}}=\frac{\delta \tau }{\varepsilon D_{s}}$$

where S and δ  are the membrane structure parameter and thickness, respectively; τ and ε are tortuosity and porosity.

The water flux across the membrane, incorporating external CP effects on the porous support layer and distinguishing feed- and draw-side mass transfer resistances, is given by [24]:

(5) $$J_{w}=A\frac{\pi_{D,b}exp\left[-J_{w}\left(\frac{1}{k_{D}}+\frac{S}{D_{D}}\right)\right]-\pi_{F,b}exp\left(\frac{J_{w}}{k_{F}}\right)}{1+\frac{B}{J_{w}}\left[exp\left(\frac{J_{w}}{k_{F}}\right)-exp\left(-J_{w}\left(\frac{1}{k_{D}}+\frac{S}{D_{D}}\right)\right)\right]}$$

where $k_{F}$ and $k_{D}$ are mass transfer coefficients at feed and dilute solutions, respectively.

For long-term FO-MD operation, water transport through the FO membrane is described using an osmotic-resistance formulation that accounts for CP and membrane fouling. Under negligible hydraulic pressure difference, the FO water flux can be expressed as [25]:

(6) $$J_{w}^{FO}=\frac{\Delta \pi -F_{cecp}\left(\pi_{F,b}+\frac{J_{s}}{J_{w}}\beta RT\right)-F_{dcp}\left(\pi_{D,b}+\frac{J_{s}}{J_{w}}\beta RT\right)}{\mu \;\left(R_{m}+R_{f}\right)}$$

where ${\Delta \pi =\pi }_{D,b}-\pi_{F,b}$ is the osmotic pressure difference between the feed and the diluted solutions, μ is the viscosity of the solvent, $R_{m}$ and $R_{f}$ represent the hydraulic resistance of the clean and the fouled membrane, respectively.

The hydraulic resistance of the clean membrane, $R_{m}$, is determined according to Darcy’s law [26]:

(7) $$A_{0}=\frac{1}{\mu R_{m}}$$

where $A_{0}$, $R_{m}$ are the water permeability coefficient and resistance of the clean FO membrane, respectively. During the concentration process, both suspended and dissolved components build up the polarization layer. From the final membrane flux ($J_{f}$), the fouling resistance, $R_{f}$, can be determined as follows [24]:

(8) $$R_{f}=\frac{\Delta P}{\mu J_{f}}-R_{m}$$

where ΔP is the transmembrane pressure difference.

The CP factors can be determined through the following equations [24]:

(9) $$F_{cecp}=\exp\left(\frac{J_{w}}{k_{cecp}}\right)-1$$

(10) $$F_{dcp}=1-\exp\left(-\frac{J_{w}}{k_{dcp}}\right)$$

Reverse salt flux, $J_{s}$, is incorporated through a constant salt permeability coefficient and expressed as [25]:

(11) $$J_{s}=-J_{w}\frac{C_{m}-C_{c}\exp\left(J_{w}S/D_{s}\right)}{\exp\left(J_{w}S/D_{s}\right)-1}$$

### 3.3. MD Transport Model

The diluted draw solution leaving the FO module serves as the feed solution to the MD module. Water transport across the hydrophobic MD membrane occurs exclusively in the vapor phase due to vapor pressure differences induced by temperature gradients.

The partial vapor pressures of water at the draw and permeate sides of the MD membrane are calculated accounting for both temperature and solution concentration effects.

(12) $$P_{v,d}=a_{w}\left(T_{d,m},C_{d}\right).P_{sat}\left(T_{d,m}\right)$$

(13) $$P_{v,p}=a_{w}\left(T_{p,m},C_{p}\right).P_{sat}\left(T_{p,m}\right)$$

where $a_{w}$ is the water activity, which accounts for the reduction in vapor pressure due to dissolved solutes, and $P_{sat}$ is the saturation vapor pressure of pure water at the given temperature.

The partial vapor pressures of water at the draw and permeate sides can be estimated using the Antoine equation [27]:

(14) $$P_{v}=\;\exp\left(23.20-\frac{3816.44}{T_{m}-46.13}\right)$$

where $T_{m}$ is the temperature at the membrane surface.

For NaCl solutions, the water activity can be estimated using the following correlation [28]:

(15) $$a_{w}=1-0.0312m-0.0014m^{2}$$

where m is the molality of the saline solution (mol·kg^−1^).

The initial MD permeate flux is described using the modified Dusty Gas Model, which takes account of the Knudsen diffusion flux, the molecular diffusion flux, and nonequimolar fluxes [29,30]:

(16) $$J_{MD,0}=\frac{\varepsilon_{MD}}{\tau_{MD}}\frac{1}{\delta_{MD}}\left(\frac{1}{\frac{1}{J_{K}}+\frac{1}{J_{M}}}\right)\left(P_{v,d}-P_{v,p}\right)$$

where:

$P_{v,d}$ and $P_{v,p}$ are the partial vapor pressures of water at the draw and permeate sides of the MD membrane, respectively.

$\varepsilon_{MD}$, $\tau_{MD}$, and $\delta_{MD}$ are the MD membrane porosity, tortuosity, and thickness, respectively.

The Knudsen diffusion flux can be written as [31]:

(17) $$J_{K}=\frac{2}{3}\frac{r_{p}}{\delta_{MD}}\sqrt{\frac{8RT}{\pi M_{w}}}$$

The molecular diffusion flux is calculated by the generalized Fick’s law [32]:

(18) $$J_{M}=\frac{D_{w,a}P}{RT\;\delta_{MD}}$$

To account for thermal inertia, draw solution concentration change, and vapor-pressure depression, the MD flux is corrected as [30]:

(19) $$J_{\mathrm{MD}}=\frac{J_{\mathrm{MD},0}}{A_{0}}exp\left[-\frac{3\;\rho_{\mathrm{MD},d}\;\;m_{s}c_{m}\;Q_{\mathrm{MD},d}}{2\;\rho_{s}\;d_{s}\;H_{\mathrm{MD},d}\;C_{T}^{2}\;A_{0}}\left(C_{L}\exp\left(\frac{C_{T}}{\eta }\left(C_{1}+\frac{t_{l}}{C_{L}}\right)\right)+\eta \;C_{T}\;C_{A}t\right)-C_{2}\right]$$

where:

$m_{s}$, $\rho_{\mathrm{MD},d}\;$ and $\rho_{s}$ are the draw mass fraction and densities of the draw solution and draw solute, respectively. $H_{\mathrm{MD},d}$, $\;d_{s}$, and $c_{m}$ are the MD draw side hydraulic diameter, draw solute diameter, friction coefficient, and coefficient of deposited mass, respectively.

$C_{T}$, $C_{L}$ and $C_{A}$ are the stress coefficient, lubrication constant, and system-dependent constant, respectively.

$C_{1}$ and $C_{2}$ are integration constants.

Heat transfer through the MD membrane is governed by conductive and convective heat fluxes [33,34]:

(20) $$q_{c}=\frac{k_{MD}}{\delta_{MD}}\left(T_{f,m}-T_{p,m}\right)$$

(21) $$q_{f}=h_{f}\left(T_{f,b}-T_{f,m}\right)$$

(22) $$q_{p}=h_{p}\left(T_{p,m}-T_{p,b}\right)$$

$k_{MD}$ is the overall mass transfer coefficient defined as [35]:

(23) $$k_{MD}=\frac{\dot{m}}{A_{m}\left(P_{f}-P_{p}\right)}$$

where $A_{m}$ is the membrane area, $P_{f}$ is the vapor pressure of the solution on the feed side of the membrane, $P_{p}$ is the vapor pressure of the condensate side, and $\dot{m}$ is the mass transfer rate.

The heat transfer coefficients $h_{f}$ and $h_{p}$ in Equations (18) and (19) depend on the flow hydrodynamics and are calculated from Nusselt number correlations. For laminar flow conditions (Re < 2100) typical in MD modules, the following correlation is used [33]:

(24) Nu=1.86Re·Pr·dhL13

For turbulent flow (Re > 2100):

(25) $$Nu=0.023{Re}^{0.8}{Pr}^{0.33}$$

where Re is the Reynolds number ($Re=\rho vd_{h}/\mu$), *Pr* is the Prandtl number, $d_{h}$ is the hydraulic diameter, and L is the channel length.

The heat transfer coefficient is then obtained from:

(26) $$h=\frac{Nu\cdot k}{d_{h}}$$

where k is the thermal conductivity of the solution.

In MD, the vaporization occurs at the hot membrane surface and the condensation occurs at the cold membrane surface, leading to the creation of a thermal boundary layer between the two membrane sides. This phenomenon is called temperature polarization, estimated using the temperature polarization coefficient (TPC), which is defined as follows:

(27) $$TCF=\frac{T_{d,m}-T_{p,m}}{T_{d,b}-T_{p,b}}$$

### 3.4. FO-MD Coupling and Water Transfer Balance

**Figure 2:**
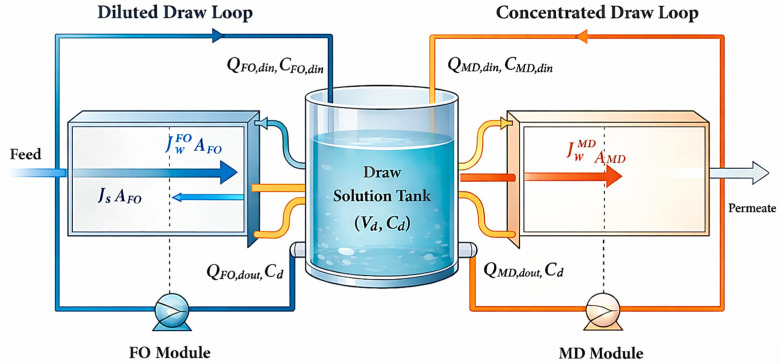
FO-MD coupling water transfer balance.

During FO-MD operation, the FO and MD modules are hydraulically and thermodynamically coupled through the draw solution, as water is transferred across the FO and MD membranes in opposite directions (Figure 2). This coupling directly affects both the volume and concentration of the draw solution and governs the overall system dynamics.

The interaction between the FO and MD modules through membrane-driven water transport can be described by a draw solution water transfer balance. The rate of change in draw solution concentration resulting solely from water permeation across the FO and MD membranes is given by:

(28) $$\frac{dC_{d}}{dt}=-\frac{J_{w}^{FO}A_{FO}}{V_{d}}C_{d}+\frac{J_{MD}A_{MD}}{V_{d}}C_{d}-\frac{J_{s}A_{FO}}{V_{d}}$$

where $C_{d}$ and $V_{d}$ are respectively the draw solution concentration and volume in the draw solution tank.

Equation (28) accounts for three phenomena affecting draw solution concentration: (1) dilution by water permeation from feed to draw across the FO membrane (first term), (2) concentration by water vapor removal across the MD membrane (second term), and (3) salt loss due to reverse salt flux across the FO membrane (third term). The RSF term is negative, indicating that salt leakage from the draw solution to the feed side reduces the draw solution concentration over time, even when water transfer rates are perfectly balanced $J_{w}^{FO}A_{FO}\;=J_{MD}A_{MD}$.

Due to the hybrid nature of the FO-MD system, the draw solution volume in the draw tank varies with time because of flow exchanges associated with both modules. Applying a volumetric balance to the draw tank yields:

(29) $$\frac{dV_{d}}{dt}=Q_{FO,din}+Q_{MD,din}-Q_{FO,dout}-Q_{MD,dout}$$

where${\;Q}_{FO,din}$ and $Q_{FO,dout}$ are the inlet and outlet flow rates of the draw solution in the FO module, respectively, while $Q_{MD,din}$ and $Q_{MD,dout}$ denote the corresponding inlet and outlet flow rates for the MD module.

The overall solute mass balance for the draw solution, accounting for inlet and outlet streams from both FO and MD modules, can be expressed as:

(30) $$\frac{dC_{d}}{dt}=\frac{1}{V_{d}}\left(C_{FO,din}Q_{FO,din}+C_{MD,din}Q_{MD,din}-C_{d}Q_{FO,dout}-C_{d}Q_{MD,dout}\right)$$

where $C_{FO,din}$ and $C_{MD,din}$ are the draw solution concentrations at the outlets of the FO and MD modules, respectively.

Equation (29) represents the general solute conservation in the draw solution tank, while Equation (30) highlights the direct FO-MD coupling through membrane water fluxes.

Under steady-state operation, the system is constrained by equal water transfer rates:

(31) $$J_{w}^{FO}A_{FO}=J_{MD}A_{MD}$$

This condition ensures constant draw solution concentration and supports stable long-term FO-MD operation. In order to solve the FO-MD mass balance equations, the values of membrane-water interface temperature on feed and permeate sides should be calculated first, by using the following equations [36]:

(32) $$T_{d,m}=\frac{\frac{\lambda_{m}}{\delta_{MD}}\left(T_{p}+\frac{h_{d}}{h_{p}}T_{d}\right)+h_{d}T_{d}-J_{MD,0}\Delta H_{v}}{\frac{\lambda_{m}}{\delta_{MD}}+h_{d}\left(1+\frac{\lambda_{m}}{\delta_{MD}h_{p}}\right)}$$

(33) $$T_{p,m}=\frac{\frac{\lambda_{m}}{\delta_{MD}}\left(T_{d}+\frac{h_{p}}{h_{d}}T_{p}\right)+h_{p}T_{p}+J_{MD,0}\Delta H_{v}}{\frac{\lambda_{m}}{\delta_{MD}}+h_{p}\left(1+\frac{\lambda_{m}}{\delta_{MD}h_{d}}\right)}$$

The synergistic coupling between FO and MD modules can be understood through the concept of dynamic equilibrium. When the system operates with $J_{w}^{FO}A_{FO}>J_{MD}A_{MD}$, the draw solution becomes diluted, reducing the osmotic driving force and consequently decreasing $J_{w}^{FO}A_{FO}$. Conversely, when $J_{w}^{FO}A_{FO}<J_{MD}A_{MD}$, the draw solution becomes concentrated, increasing the osmotic driving force and enhancing $J_{w}^{FO}A_{FO}$. This negative feedback mechanism naturally drives the system toward an equilibrium state where the water transfer rates balance according to Equation (31). The characteristic time required to reach this equilibrium depends on the drawing volume, membrane areas, and the sensitivity of flux to concentration changes.

The physicochemical properties of the FO and MD membranes used in this study are summarized in Table 1 [37,38]. In addition, detailed documentation of all membrane parameters and their corresponding literature sources is provided in Table 2.

The initial conditions used for each pre-balancing test are given in Table 3 [30]. The conditions used for seawater desalination simulations are presented in Table 4.

## 4. Results and Discussion

### 4.1. Model Validation

**Figure 3:**
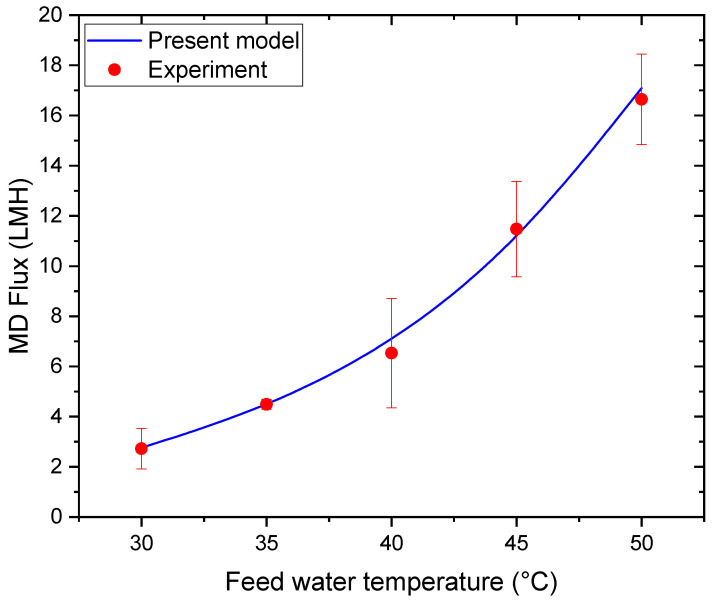
Variation in MD permeate flux with feed temperature.

The mathematical model developed in this study was validated against experimental data reported by Zohrabian et al. [30]. Figure 3 compares the MD permeate fluxes measured for a 0.5 M NaCl solution at feed temperatures ranging from 30 to 50 °C with the corresponding predictions of the present numerical model. As shown in this figure, the MD permeate flux increases significantly with increasing feed water temperature over the investigated range. This trend is attributed to the exponential increase in water vapor pressure at the membrane-feed interface with temperature, which enhances the vapor pressure gradient driving mass transfer across the hydrophobic membrane.

The close agreement between the experimental data and the present model indicates that the model accurately captures the thermodynamic dependence of vapor transport, including the effects of temperature polarization. At higher temperatures, slight deviations between modeled and experimental fluxes may be associated with increased conductive heat losses and intensified temperature polarization, phenomena commonly reported in MD systems operating under elevated thermal gradients. Similar temperature-dependent flux behavior has been widely documented in the MD literature and confirms that feed temperature is a dominant operational parameter governing MD performance [33,40,41,42,43].

### 4.2. Sensitivity Analysis

To assess the robustness of model predictions, we conducted a sensitivity analysis examining the impact of uncertainties in key polarization parameters on predicted fluxes. The analysis focused on three parameters: the mass transfer coefficient (*k*) affecting concentration polarization in FO, and the heat transfer coefficients ($h_{f}$, $h_{p}$) affecting temperature polarization in MD.

The results (Table 5) show that FO flux is moderately sensitive to mass transfer coefficients, with a 20% variation in k causing 6–9% changes in predicted flux. This sensitivity arises because concentration polarization reduces the effective osmotic pressure difference, and inaccurate k values lead to incorrect estimation of this reduction. The slightly higher sensitivity to $k_{f}$ compared to $k_{D}$ reflects the asymmetric structure of FO membranes.

MD flux shows lower sensitivity to heat transfer coefficients (3–5% change for 20% parameter variation), indicating that temperature polarization, while significant, is less critical than the exponential dependence on bulk temperature. The higher sensitivity to $h_{f}$ compared to $h_{p}$ is expected because the feed side temperature drives the vapor pressure gradient.

Overall, the model predictions are moderately sensitive to polarization parameters, with FO showing greater sensitivity than MD. This underscores the importance of using accurate correlations for mass and heat transfer coefficients and suggests that experimental characterization of these parameters in specific module geometries would improve predictive accuracy. However, even with the uncertainties examined, the qualitative trends and relative comparisons presented in this study remain valid.

**Figure 4:**
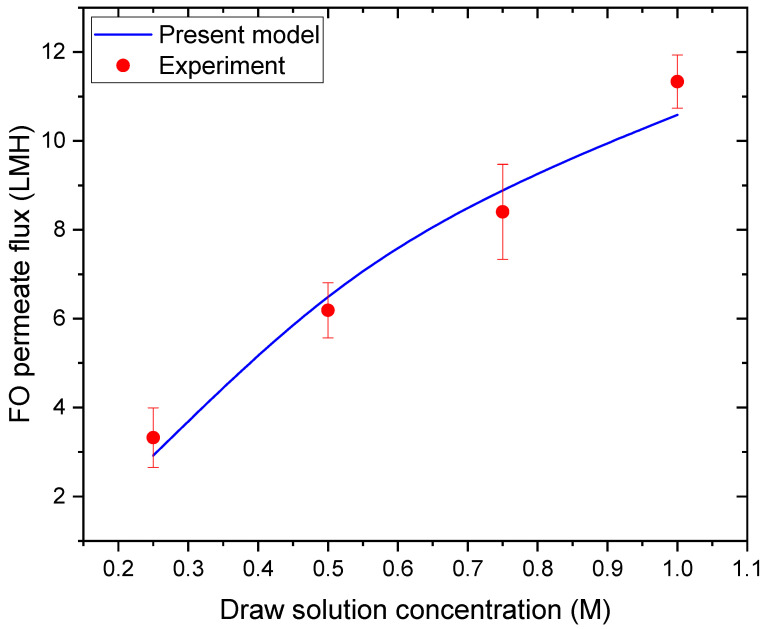
Variation in FO permeate flux with draw solution concentration.

Figure 4 illustrates the variation in FO permeate flux as a function of raw solution concentration. An increase in draw solution concentration from 0.2 to 1.1 M results in a pronounced increase in FO flux, as shown in Figure 4. This behavior is primarily due to the enhancement of the osmotic pressure gradient across the FO membrane when the draw solution concentration increases, while the feed solution (deionized water) maintains a constant osmotic pressure near zero. The increase in osmotic pressure difference outweighs the adverse effects of concentration polarization within the examined concentration range, resulting in a net increase in water flux.

The strong correspondence between experimental measurements and model predictions demonstrates that the model effectively incorporates osmotic pressure variation and CP effects. These observations are consistent with classical FO transport theory and previously reported experimental studies, which indicate that feed salinity exerts a substantial influence on FO water flux [44,45].

**Figure 5:**
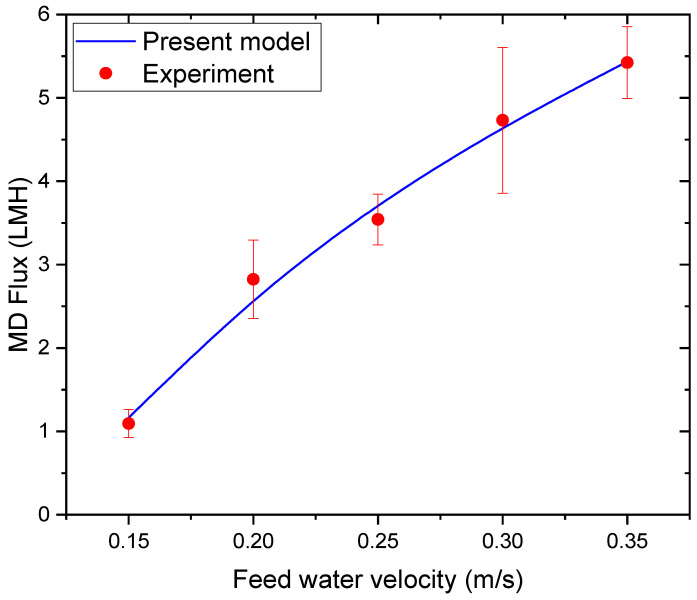
Variation in MD permeate flux with feed cross-flow velocity.

The influence of feed cross-flow velocity on MD permeate flux is presented in Figure 5. Increasing the feed velocity from 0.15 to 0.35 m/s leads to a measurable improvement in permeate flux. This improvement is attributed to the reduction in thermal boundary layer thickness and the mitigation of temperature polarization at higher flow velocities. Enhanced convective heat transfer maintains a higher effective membrane surface temperature, thereby sustaining a larger vapor pressure gradient across the membrane.

However, the rate of flux increase diminishes at higher velocities, suggesting that membrane mass transfer resistance becomes the controlling factor once hydrodynamic limitations are reduced. The agreement between the experimental data and the model confirms the ability of the model to accurately represent hydrodynamic effects in MD operation.

**Figure 6:**
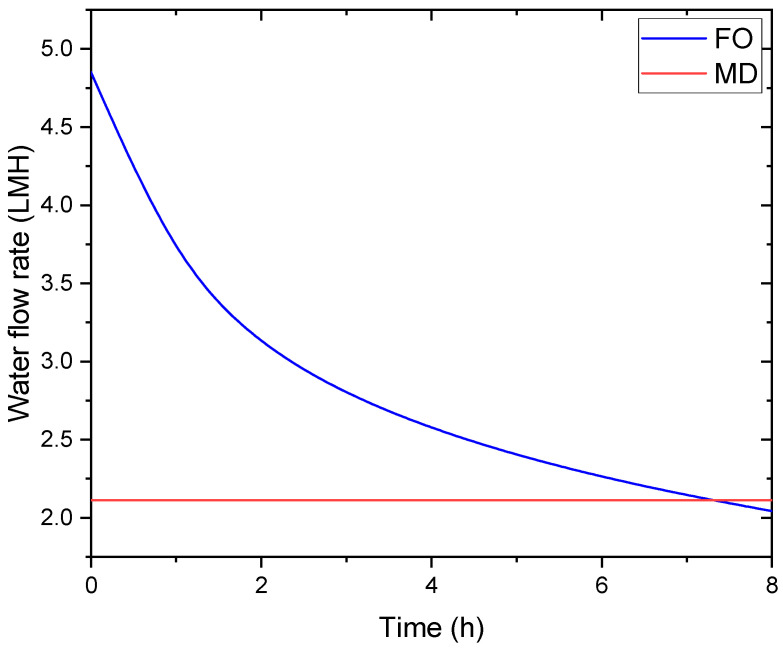
Time-dependent water fluxes in the integrated FO-MD system.

Figure 6 shows the temporal evolution of water fluxes in the coupled FO-MD system under the conditions specified in Table 3 (seawater feed). Initially, FO water flux (4.85 LMH) exceeds MD flux (2.11 LMH), meaning that water enters the draw solution loop from the feed faster than it is removed by MD. This imbalance causes progressive dilution of the draw solution, which is reflected in the declining FO flux over time; from 4.85 LMH initially to 2.4 LMH after 5 h (a 50% decrease).

In a standalone FO system, the FO flux would decline by approximately 70% over the same period due to unmitigated draw dilution. The MD module, by continuously removing water vapor from the draw solution at an average rate of 2.11 LMH over the 8 h period, reduces the net rate of draw solution dilution by approximately 60%.

Thus, while MD does not completely eliminate draw dilution, it substantially slows the dilution rate and enables the system to approach a stable operating point that would be impossible without regeneration. This dynamic interaction highlights the synergistic nature of the FO-MD hybrid configuration, which allows sustained water production by coupling osmotic-driven and thermally driven processes and separations mechanisms. Similar time-dependent flux behavior has been reported in previous FO-MD hybrid studies [20,44].

**Figure 7:**
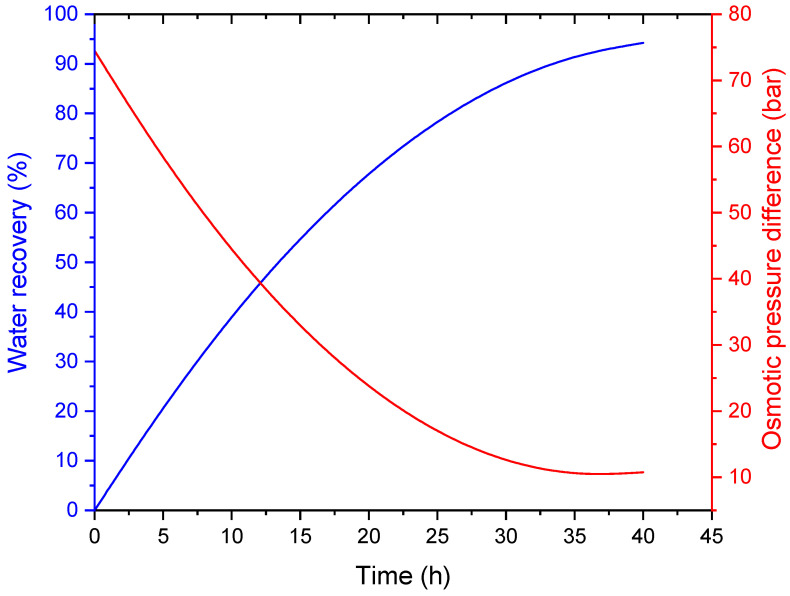
Variation in FO-MD water recovery and osmotic pressure with time.

Figure 7 illustrates the evolution of water recovery and osmotic pressure difference as a function of operating time. Water recovery increases steadily, indicating continuous permeation of water through the FO membrane. Concurrently, the osmotic pressure difference decreases due to dilution of the draw solution. The relatively gradual decline in osmotic pressure suggests effective draw solution reconcentration by the MD process, which helps maintain a sufficient driving force for FO operation.

This inverse relationship between water recovery and osmotic pressure difference underscores the importance of draw solution regeneration in achieving high recovery ratios in FO-based systems. The observed trends confirm the suitability of the FO-MD hybrid approach for long-term operation with improved overall water recovery.

**Figure 8:**
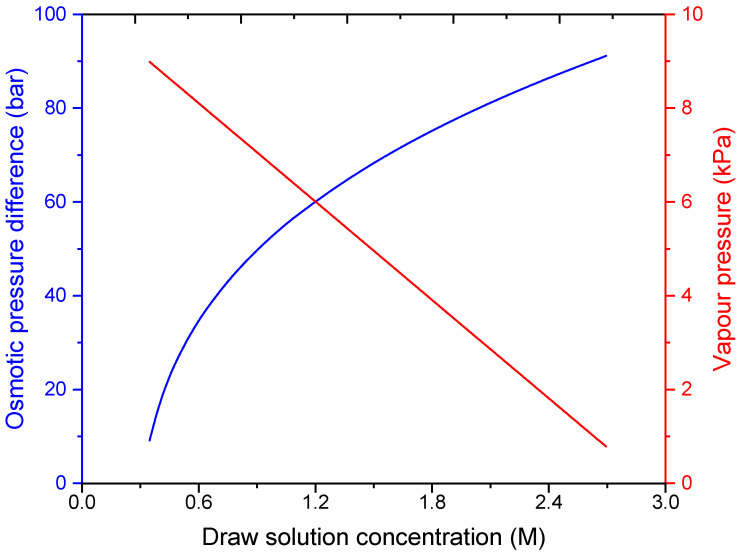
Variation in osmotic pressure difference and water vapor pressure at the MD membrane surface with draw solution concentration.

Figure 8 illustrates the contrasting sensitivities of FO and MD processes to draw solution concentration. For FO, the osmotic pressure difference (Δπ) increases sharply as draw concentration increases, because $\pi_{D,b}$ rises while $\pi_{F,b}$ is held constant at 0.6 M (seawater). This explains why FO performance improves at higher draw concentrations (the driving force is enhanced). For MD, the vapor pressure shown is that of the draw solution entering the MD module. As draw concentration increases, the vapor pressure decreases only slightly due to the vapor pressure depression effect of dissolved salts. Consequently, the MD driving force is relatively insensitive to draw concentration across the investigated range.

This fundamental difference in concentration sensitivity is a key advantage of the hybrid FO-MD configuration: FO benefits strongly from a highly concentrated draw solution, while MD can effectively regenerate that draw solution without being substantially hindered by the very high concentrations that favor FO. The thermal driving force in MD remains robust across a wide range of draw concentrations, whereas the osmotic driving force in FO is highly concentration dependent.

**Figure 9:**
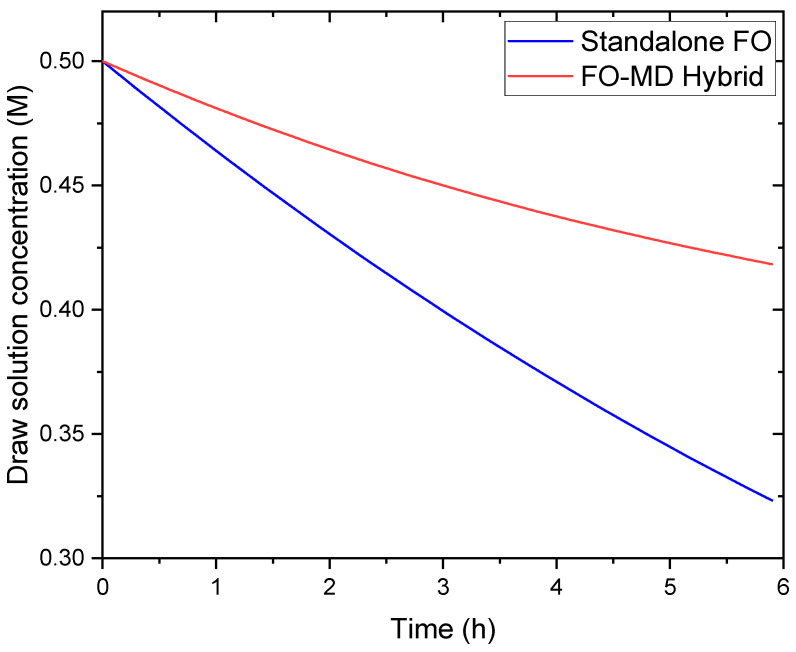
Evolution of draw solution concentration over time with and without MD regeneration.

To quantitatively demonstrate the synergistic effect of FO-MD coupling, we conducted comparative simulations of the draw solution concentration evolution with and without MD regeneration (Figure 9). In the standalone FO configuration (without MD regeneration), the draw solution concentration decreases exponentially from an initial 0.5 M to approximately 0.32 M after 6 h of operation, representing a 36% dilution. This dilution reduces the osmotic pressure difference by approximately 45%, causing a corresponding decline in FO water flux.

In contrast, in the integrated FO-MD system, the draw solution concentration decreases more gradually, reaching only 0.42 M after 6 h (a 14% dilution). The MD module, operating with a feed temperature of 50 °C, reconcentrates the draw solution by extracting water vapor at a rate of 2.16 LMH, which partially offsets the dilution caused by FO water permeation (initial flux 4.8 LMH). This dynamic balance maintains the osmotic pressure difference above 75% of its initial value throughout the 6 h operation.

The synergy can be quantified by the regeneration efficiency, defined as the ratio of MD water removal rate to FO water permeation rate. Under the conditions studied, this ratio ranges from 0.45 (initially) to 0.85 (after 6 h), indicating that MD increasingly compensates for FO dilution as the system approaches steady state. This regenerative coupling enables sustained FO performance without the need for chemical replenishment of the draw solution, which is a key limitation of standalone FO systems.

### 4.3. Reverse Salt Flux Impacts

RSF is an inherent phenomenon in FO processes where draw solutes diffuse back across the membrane into the feed side due to the concentration gradient. In the FO-MD hybrid system, RSF has several important implications for long-term performance:

#### 4.3.1. Draw Solution Depletion

As shown in the revised Equation (21), RSF directly reduces draw solution concentration at a rate proportional to $J_{s}=A_{FO}/V_{d}$. For the membrane used in this study (B=0.09 LMH) and operating conditions in Table 3, the cumulative salt loss after 24 h of operation is approximately 0.12 mol, representing a 8% reduction in draw solution concentration (from 1.5 M to 1.38 M) that cannot be recovered by MD (since MD retains all non-volatile solutes). This loss must be compensated by periodic draw solute replenishment.

#### 4.3.2. Feed Salinity Increase

RSF increases the feed solution salinity over time, which reduces the net osmotic driving force. After 24 h, the feed concentration increases from 0.6 M to approximately 0.65 M due to RSF, causing a 5–7% reduction in FO flux beyond the effect of draw dilution alone.

#### 4.3.3. Enhanced Fouling/Scaling Potential

The accumulation of draw solutes (typically NaCl) in the feed side can exacerbate scaling on the FO membrane feed surface, particularly if the feed contains multivalent ions that form precipitates with chloride.

#### 4.3.4. Membrane Wetting Risk on MD Side

While not directly affecting the MD membrane, RSF reduces draw solution concentration, which slightly increases the vapor pressure (by ~2% for a 0.12 M concentration decrease). This marginally enhances MD flux but at the cost of reduced FO driving force leading to a trade-off that must be carefully optimized at system level.

#### 4.3.5. Long-Term Sustainability

The irreversible nature of salt loss means that standalone FO-MD systems cannot operate indefinitely without draw solute replenishment. The replenishment frequency depends on the ratio of draw solution volume to membrane area ($V_{d}/A_{FO}$) and the salt permeability coefficient B. For the laboratory-scale system studied ($V_{d}/A_{FO}\approx 240\;$ L/m^2^), replenishment would be needed every 3–5 days of continuous operation. For larger systems, proportionally larger draw tanks or continuous dosing systems would be required.

These impacts highlight the importance of developing FO membranes with low salt permeability (B <0.05 LMH) and exploring draw solutes with larger molecular sizes that exhibit lower reverse diffusion. Future work should investigate the techno-economic optimization of replenishment frequency versus membrane replacement costs.

### 4.4. Energy Efficiency Analysis

To quantify the energy efficiency benefits of the FO-MD hybrid system, we calculated the specific energy consumption (SEC) and compared it with standalone FO and MD systems for seawater desalination (Table 6).

The obtained results demonstrate that FO-MD hybridization offers meaningful energy savings compared to standalone thermal processes, while enabling higher water recovery than pressure-driven processes for high-salinity feeds. However, SEC remains substantially higher than RO for seawater desalination, indicating that FO-MD is most suitable for niche applications such as high-salinity brine concentration, industrial wastewater treatment, or scenarios with abundant low-grade waste heat.

### 4.5. Scalability and Practical Challenges

The FO-MD hybrid system presented in this study was evaluated at laboratory scale (membrane areas < 0.01 m^2^). Translating this concept to practical applications requires consideration of several scalability factors and operational challenges.

#### 4.5.1. Scalability Assessment

(a) Membrane module configuration: The current configuration uses separate membrane modules for FO and MD, which is readily scalable by increasing membrane area through standard module designs (spiral-wound for FO, hollow-fiber for MD). Commercial FO elements are available up to 40 m^2^, and MD modules up to 10 m^2^, allowing straightforward scale-up to pilot scale (1–10 m^3^/d). Further scale-up to industrial scale (100–1000 m^3^/d) would require multiple modules in parallel arrays, which is standard practice in membrane processes.

(b) Draw solution volume: The draw tank volume must scale with membrane area to maintain stable operation. The characteristic time constant ($\tau =V_{d}/A_{FO}\times dJ_{w}/dC$) should be >1 h to allow stable control. For a 100 m^3^/d plant (FO area ≈ 2000 m^2^,$\;J_{w}$ ≈ 5 LMH), this requires $V_{d\;}\approx \;10-20$ m^3^, which is manageable.

(c) Heat integration: At larger scales, heat recovery becomes economically attractive. The latent heat of condensation (≈2257 kJ/kg permeate) can be recovered to preheat the MD feed stream, potentially reducing thermal energy consumption by 40–50%, as shown in Table 5.

#### 4.5.2. Practical Challenges for Real Seawater Operation

(a) Membrane fouling:

- FO membrane: Seawater contains organic matter, colloids, and biological organisms that cause fouling. Our model includes a fouling resistance term ($R_{f}$), which would increase over time. For real seawater, $R_{f}$ could increase by 0.5–2 × 10^13^ m^−1^ per month, requiring periodic cleaning. Pretreatment (microfiltration/ultrafiltration) would be essential to reduce fouling rates.
- MD membrane: Scaling by calcium sulfate or calcium carbonate is a major concern due to the elevated temperatures. Antiscalants or periodic acid cleaning would be necessary.

(b) Draw solution management:

- Solute selection: NaCl was used in this study, but alternative draw solutes (e.g., MgCl_2_, CaCl_2_, or thermolytic salts like NH_4_HCO_3_) could offer lower reverse salt flux or easier regeneration.
- Replenishment: As discussed earlier, RSF requires periodic draw solute addition. For a 100 m^3^/d plant with B = 0.09 LMH, daily salt loss would be ≈180 kg NaCl, representing a significant operating cost.
- Concentration control: Maintaining optimal draw concentration requires feedback control of MD operating temperature or flow rates to balance FO and MD fluxes.

(c) Temperature control: Seasonal seawater temperature variations (5–30 °C) affect FO flux (through osmotic pressure temperature dependence) and the heating requirement for MD. In winter, additional heating may be needed; in summer, cooling water availability for permeate condensation may be limited.

(d) Membrane wetting: Long-term MD operation risks pore wetting, especially if surfactants or amphiphilic compounds are present in the feed. Membrane development with enhanced hydrophobicity or omniphobic coatings would improve robustness.

(e) Energy source integration: The economic viability depends on access to low-grade waste heat (60–80 °C) from industrial processes, solar thermal collectors, or geothermal sources. Without such heat sources, the thermal energy cost would be prohibitive.

#### 4.5.3. Mitigation Strategies

- Pretreatment: MF/UF pretreatment for FO feed to remove suspended solids and large organic molecules.
- Antiscalants: Dosing of scale inhibitors for MD feed.
- Periodic cleaning: CIP (clean-in-place) protocols for both membranes.
- Advanced draw solutes: Investigation of polyelectrolytes or switchable solvents with lower reverse flux.
- Heat recovery: Implementation of plate heat exchangers to recover latent heat.
- Hybrid operation: Occasional flushing of FO feed side with low-salinity water to remove accumulated salts from reverse flux.

These considerations suggest that FO-MD hybrid systems are technically scalable but face significant operational challenges that require further research and development before widespread commercial deployment for seawater desalination. Near-term applications may focus on industrial wastewater treatment or brine concentration where the benefits justify the complexity.

### 4.6. Comparative Study

To contextualize the findings of this study within the broader literature, we present a comparative analysis of FO-MD hybrid system performance reported in recent studies (Table 7).

As shown in this table, the FO fluxes predicted in this study (5.05 LMH) fall within the range reported in experimental studies (2.8–9.2 LMH), confirming that the model produces realistic values. MD fluxes (2.50 LMH) are at the lower end of reported ranges, reflecting the moderate operating temperature (50 °C) and seawater feed salinity.

It can also be seen that most literature studies focus on wastewater treatment (landfill leachate, produced water) where FO-MD advantages are clear. This study extends analysis to seawater desalination, a more challenging application due to the lower value of product water and strong competition from nature RO technology.

Finally, unlike purely experimental studies, this work provides a validated modeling framework that can be used for system optimization and scale-up without extensive experimental campaigns.

## 5. Conclusions

In this study, a comprehensive modeling framework was developed to analyze coupled heat, mass, and solute transport in a hybrid FO-MD system for seawater desalination, with several novel contributions to the understanding of these systems. The proposed FO-MD model was validated against experimental data for both FO and MD modules independently, establishing confidence in the coupled system predictions. The main findings can be summarized as follows:

- The MD permeate flux increased from approximately 2–3 LMH at 30 °C to 17 LMH at 50 °C, while FO flux increased with draw solution concentration from 0.2 to 1.1 M, confirming proper representation of the respective driving forces.
- Time-dependent simulations revealed that MD regeneration reduces draw solution dilution by 60% compared to standalone FO operation, maintaining FO flux 43% higher after 6 h. The regeneration efficiency (ratio of MD water removal to FO permeation) increases from 0.45 to 0.85 as the system approaches dynamic equilibrium, quantitatively demonstrating the synergistic benefit.
- The cumulative salt loss reaches 8% of draw solution concentration after 24 h, requiring periodic replenishment. This irreversible loss represents a fundamental limitation of FO-MD systems that must be considered in long-term operation.
- FO flux predictions show moderate sensitivity (6–9% change for 20% parameter variation) to mass transfer coefficients affecting concentration polarization, while MD flux is less sensitive (3–5%) to heat transfer coefficients. These findings identify key parameters requiring accurate characterization for reliable modeling.
- FO-MD hybridization reduces thermal energy consumption by 15–40% compared to standalone MD, with specific energy consumption of 382 kWh/m^3^ (40.2 kWh/m^3^ primary energy equivalent) when using low-grade heat. With heat recovery, thermal SEC could potentially be reduced by 50%, approaching the efficiency of pressure-driven processes for suitable applications.
- Osmotic pressure increases sharply with feed concentration, reducing FO driving force, while vapor pressure decreases only gradually, maintaining MD performance. This fundamental difference explains the advantage of MD for draw solution regeneration in high-salinity environments and justifies the hybrid approach.
- Laboratory-scale results were contextualized for potential scale-up, identifying key challenges including membrane fouling, draw solute replenishment, temperature control, and membrane wetting. Mitigation strategies including pretreatment, antiscalants, advanced draw solutes, and heat recovery were proposed.

Overall, the obtained results demonstrate that the hybrid FO-MD system combines the high osmotic driving force of FO with the thermal robustness of MD, enabling higher water recovery, stabilized long-term flux, and effective draw solution management compared to standalone processes. However, future studies should focus on long-term experimental operation under real seawater conditions to assess fouling, scaling, and membrane wetting phenomena. The performance and stability of alternative draw solutions with reduced reverse salt flux should also be investigated. In addition, the integration with renewable or waste heat sources should be explored to quantify realistic energy savings. Finally, extending the present model to multidimensional CFD simulations would allow detailed visualization of local concentration and temperature gradients, providing deeper insight into polarization effects and supporting further system optimization.

## Figures and Tables

**Table 1:** Key properties of FO and MD membranes [37,38].

	Length (m)	Width (m)	Thickness (μm)	Pore Size (nm)	Water Permeability Coefficient, *A* (LMH/Bar)	Solute Permeability Coefficient, *B* (LMH)	Structural Parameter, *S* (μm)
FO	0.1015	0.041	110	–	0.52 ± 0.06	0.09	630
MD	0.09	0.04	160	220	–	–	–

**Table 2:** Detailed MD membrane parameters and literature sources.

Parameter	Value	Source	Notes
Membrane material	PTFE	Manufacturer [38]	Hydrophobic, chemically resistant
Nominal pore size	220 nm	Manufacturer [38]	As specified in product datasheet
Thickness ($\delta_{MD}$)	160 μm	Manufacturer [38]	Total thickness including support
Porosity ($\varepsilon_{MD}$)	0.75	Estimated from manufacturer data	Typical range 0.70–0.80 for PTFE membranes
Tortuosity ($\tau_{MD}$)	1.8	Calculated from $\varepsilon_{MD}$ using correlation $\tau =\left(2-\varepsilon_{MD}\right)/\varepsilon_{MD}$ [39]	Consistent with literature values for similar membranes
Thermal conductivity ($k_{m}$)	0.25 W/m K	[33,40]	For dry PTFE; the effective value would be higher when wetted
Water contact angle	135°	Manufacturer [38]	Confirms hydrophobicity
Liquid entry pressure (LEP)	2.5 bar	Manufacturer [38]	Maximum pressure before pore wetting

**Table 3:** Initial conditions applied to the FO-MD hybrid system [30].

Parameter	Value
Feed tank concentration (M)	0
Draw tank concentration (M)	0.5
Feed tank volume (L)	0.7
Draw tank volume (L)	0.4
Permeate tank volume (L)	0.4
FO permeate flux (LMH)	6.17
MD permeate flux (LMH)	3.14

**Table 4:** Operating conditions for seawater desalination simulations.

Parameter	Value
Feed concentration (seawater)	0.6 M NaCl
Draw solution initial concentration	1.5 M NaCl
Feed temperature	25 °C
MD feed temperature	50 °C
Permeate temperature	20 °C
Feed tank volume	2.0 L
Draw tank volume	1.0 L
Permeate tank volume	1.0 L
Cross-flow velocity (both modules)	0.25 m/s

**Table 5:** Sensitivity of model predictions to ±20% variations in key parameters.

Parameter	Baseline Value	Variation	FO Flux Change	MD Flux Change
$k_{f}$ (FO feed side mass transfer coeff.)	2.5 × 10^−5^ m/s	+20%	+7.2%	—
		−20%	−8.5%	—
$k_{D}$ (FO draw side mass transfer coeff.)	2.5 × 10^−5^ m/s	+20%	+5.8%	—
		−20%	−6.9%	—
$h_{f}$ (MD feed side heat transfer coeff.)	2500 W/m^2^ K	+20%	—	+4.3%
		−20%	—	−5.1%
$h_{p}$ (MD permeate side heat transfer coeff.)	2000 W/m^2^ K	+20%	—	+3.2%
		−20%	—	−3.8%

**Table 6:** Comparison of specific energy consumption (kWh/m^3^ of permeate) for different configurations [15,33].

Configuration	Thermal Energy	Electrical Energy	Total SEC	Primary Energy Equivalent *
Standalone FO (with external DS regeneration)	0	8.5 (regeneration not included)	>8.5 **	>8.5
Standalone MD	450–650	1.2–1.8	450–650	45–65 ***
FO-MD hybrid (this study)	380	2.1	382.1	40.2
FO-MD with heat recovery (projected)	190	2.1	192.1	21.2
RO (reference)	0	3.5–4.5	3.5–4.5	3.5–4.5

* Primary energy equivalent converts thermal energy to equivalent electrical energy using a 40% conversion efficiency for comparison purposes. ** Standalone FO requires a separate draw solution regeneration process (typically RO or thermal), which dominates energy consumption. *** Assuming thermal energy from low-grade waste heat with zero marginal cost would reduce effective SEC.

**Table 7:** Comparison of FO-MD hybrid system performance with literature studies.

Study	Feed Type	FO Membrane	MD Membrane	FO Flux (LMH)	MD Flux (LMH)	Key Findings
This study (model)	Seawater (0.6 M NaCl)	CTA (A = 0.52 LMH/bar)	PTFE (0.22 μm)	5.05	2.50	Quantified synergy (60% dilution reduction); SEC = 382 kWh/m^3^
Zohrabian et al. [30] (experimental)	DI water/NaCl solutions	CTA (Aquaporin)	PTFE (0.22 μm)	6.17	3.14	Demonstrated technical feasibility of FO-MD coupling
Zhou et al. [18] (experimental)	Landfill leachate	CTA (HTI)	PVDF (0.22 μm)	4.2–8.5	2.8–5.6	Optimized operating parameters; achieved >99% contaminant rejection
Nawaz et al. [17] (experimental)	Produced water	TFC (various)	PTFE (0.45 μm)	3.5–7.2	2.1–4.8	Evaluated real produced water; reported fouling behavior
Mat Nawi et al. [20] (experimental)	Produced water	CTA (HTI)	PVDF (0.22 μm)	2.8–6.5	1.9–4.2	Studied flux dynamics and FO-MD equilibrium
Kim et al. [40] (experimental)	Synthetic seawater	TFC (Toray)	PVDF (0.45 μm)	4.8–9.2	3.5–6.8	Developed thermally isolated integrated module
Ricci et al. [46] (experimental)	Synthetic wastewater	CTA (HTI)	PTFE (0.22 μm)	3.2–5.8	2.2–4.5	Assessed submerged hybrid system performance

## Data Availability

Will be available after acceptance and publication in this journal.

## Nomenclature

### Abbreviations

**Latin Symbols**		
**Symbol**	**Definition**	**Unit**
A	FO membrane water permeability coefficient	LMH·bar^−1^
$A_{0}$	Water permeability coefficient of clean membrane	LMH·bar^−1^
$A_{m}$	Membrane area	m^2^
$A_{FO}$	FO membrane area	m^2^
$A_{MD}$	MD membrane area	m^2^
$a_{w}$	Water activity	–
B	FO membrane solute permeability coefficient	LMH
C	Molar concentration	mol·L^−1^
$C_{d}$	Draw solution concentration	mol·L^−1^
$C_{1}$,$C_{2}$	Integration constants	–
$C_{T}$	Stress coefficient	–
$C_{L}$	Lubrication constant	–
$C_{A}$	System-dependent constant	–
$c_{m}$	Coefficient of deposited mass	–
$D_{s}$	Solute diffusion coefficient	m^2^·s^−1^
$D_{w,a}$	Water-air diffusion coefficient	m^2^·s^−1^
$d_{h}$	Hydraulic diameter	M
$d_{s}$	Draw solute diameter	M
$F_{cerp}$	Concentration polarization factor (feed side)	–
$F_{dcp}$	Concentration polarization factor (draw side)	–
f	Friction coefficient	–
$H_{MD,d}$	MD draw side hydraulic diameter	M
$h_{d}$,$h_{p}$,$h_{f}$	Heat transfer coefficients	W·m^−2^·K^−1^
$J_{w}$	Water flux	LMH or m^3^·m^−2^·s^−1^
$J_{w}^{FO}$	FO water flux	LMH
$J_{MD}$	MD water flux	LMH
$J_{MD,0}$	Initial MD flux	LMH
$J_{s}$	Reverse salt flux	mol·m^−2^·s^−1^
$J_{K}$	Knudsen diffusion flux	kg·m^−2^·s^−1^
$J_{M}$	Molecular diffusion flux	kg·m^−2^·s^−1^
k	Mass transfer coefficient	m·s^−1^
$k_{F}$, $k_{D}$	Mass transfer coefficients (feed/draw)	m·s^−1^
$k_{MD}$	Thermal conductivity of MD membrane	W·m^−1^·K^−1^
K	Solute resistivity	s·m^−1^
L	Channel lenght	m
$M_{w}$	Molecular weight of water	kg·mol^−1^
$\dot{m}$	Mass transfer rate	kg·s^−1^
$m_{s}$	Mass fraction of draw solute	–
n	van’t Hoff coefficient	–
Nu	Nusselt number	–
P	Vapor pressure	Pa
$P_{sat}$	Saturation vapor pressure	Pa
$P_{v}$	Partial vapor pressure of water	Pa
$P_{v,d}$, $P_{v,p}$	Vapor pressure on draw/permeate side	Pa
Pr	Prandtl number	–
ΔP	Transmembrane pressure difference	Pa
Q	Volumetric flow rate	m^3^·s^−1^
$Q_{FO,din}$, $Q_{FO,dout}$	FO inlet/outlet draw flow rates	m^3^·s^−1^
$Q_{MD,din}$, $Q_{MD,dout}$	MD inlet/outlet draw flow rates	m^3^·s^−1^
$q_{c}$, $q_{f},\;q_{p}$	Conductive, feed-side, permeate-side heat fluxes	W·m^−2^
R	Ideal gas constant	J·mol^−1^·K^−1^
$R_{m}$	Hydraulic resistance of clean membrane	m^−1^
$R_{f}$	Fouling resistance	m^−1^
Re	Raynolds number	–
$r_{p}$	Pore radius (MD membrane)	m
S	Structural parameter of FO membrane	m
Sh	Sherwood number	–
T	Temperature	K
$T_{m}$	Temperature at membrane surface	K
$T_{d,m}$, $T_{p,m}$	MD draw/permeate side membrane temperature	K
$T_{f,b}$, $T_{p,b}$	Bulk feed/permeate temperature	K
t	Time	s
$t_{l}$	Time-related constant	s
V	Volume	m^3^
$V_{d}$	Draw tank volume	m^3^
v	Cross-flow velocity	m·s^−1^
**Greek Symbols**		
**Symbol**	**Definition**	**Unit**
α	van’t Hoff coefficient	–
β	Constant related to reverse salt flux	–
γ	System-dependent constant	–
Δ	Difference (driving force)	–
δ	Membrane thickness	m
$\delta_{MD}$	MD membrane thickness	m
ε	Membrane porosity	–
$\varepsilon_{MD}$	MD membrane porosity	–
η	Viscosity-related constant	–
λ	Lubrication constant	–
$\lambda_{m}$	Membrane thermal conductivity	W·m^−1^·K^−1^
μ	Dynamic viscosity	Pa·s
π	Osmotic pressure	Pa
$\pi_{D,b}$, $\pi_{F,b}$	Bulk osmotic pressure (draw/feed)	Pa
ρ	Density	kg·m^−3^
$\rho_{s}$	Draw solute density	kg·m^−3^
$\rho_{MD,d}$	MD draw solution density	kg·m^−3^
σ	Stress coefficient	–
τ	Membrane tortuosity	–
ϕ	Osmotic coefficient	–
$\tau_{MD}$	MD membrane tortuosity	–
${\Delta H}_{v}$	Latent heat of vaporization	J·kg^−1^
**Subscripts**		
**Subscript**	**Meaning**	
b	Bulk solution	
c	Condensate	
d	Draw solution	
f	Feed solution	
FO	Forward osmosis	
MD	Membrane distillation	
m	Membrane	
p	Permeate	
s	Solute	
v	Vapor	
w	Water	
**Abbreviations**		
**Abbreviation**	**Meaning**	
CP	Concentration polarization	
DCMD	Direct contact membrane distillation	
FO	Forward osmosis	
LMH	L·m^−2^·h^−1^	
MD	Membrane distillation	
PRO	Pressure retarded osmosis	
RSF	Reverse salt flux	
TPC	Temperature polarization coefficient	
